# Dislocated Galeazzi-Equivalent Fracture With Ulnar Physeal Injury in an 11-Year-Old Boy

**DOI:** 10.7759/cureus.75921

**Published:** 2024-12-18

**Authors:** Patrik Cervenak, Birte Schultz, Stefan Dierauer

**Affiliations:** 1 Orthopaedics and Traumatology, Orthopaedic Hospital Sonnenhof, Bern, CHE

**Keywords:** distal radioulnar joint (druj), galeazzi-equivalent fracture, osteosynthesis, trauma pediatric, ulnar physeal fracture

## Abstract

Galeazzi-equivalent fracture is a very rare type of fracture. Unlike the "classic" adult form of Galeazzi fracture, where the distal radioulnar joint (DRUJ) is dislocated and the triangular fibrocartilage complex (TFCC) is often damaged, the DRUJ and TFCC may remain intact in children. In this article, we report the case of an 11-year-old boy with a Galeazzi-equivalent fracture. Awareness of this kind of injury is important to prevent malreduction of the ulna.

## Introduction

The “classic” Galeazzi fracture is an injury involving a fracture of the radial shaft and the injury of the distal radioulnar joint (DRUJ). Fractures in the pediatric population affect the growth plate in approximately 15-18% of cases [1]. Galeazzi-equivalent fractures are unique to the pediatric population. The distal radial shaft fracture is associated with disruption of the distal ulnar physis. In contrast to the “classic” Galeazzi fracture where the DRUJ is dislocated and the triangular fibrocartilage complex (TFCC) is injured, the DRUJ and TFCC can remain intact in pediatric cases. Instead, the deforming force typically dislocates the distal ulnar growth plate. It is estimated that approximately 81% of longitudinal ulnar growth occurs at the distal ulnar growth plate [2]. While the distal radial fracture component is clearly visible on X-rays, the disruption of the distal ulnar physis can easily be overlooked. Disruptions to the distal ulnar physis can result in growth arrest and forearm deformity, which can also alter wrist kinematics. In this article, we report the case of an 11-year-old boy with a Galeazzi-equivalent fracture. After an unsuccessful closed reduction, an open reposition and internal fixation were necessary. Written informed consent for publication of this case was provided by the patient's parents. This case was previously presented as a poster at the "82. Jahreskongress swiss orthopedics" on June 23, 2022.

## Case presentation

**Figure 1:**
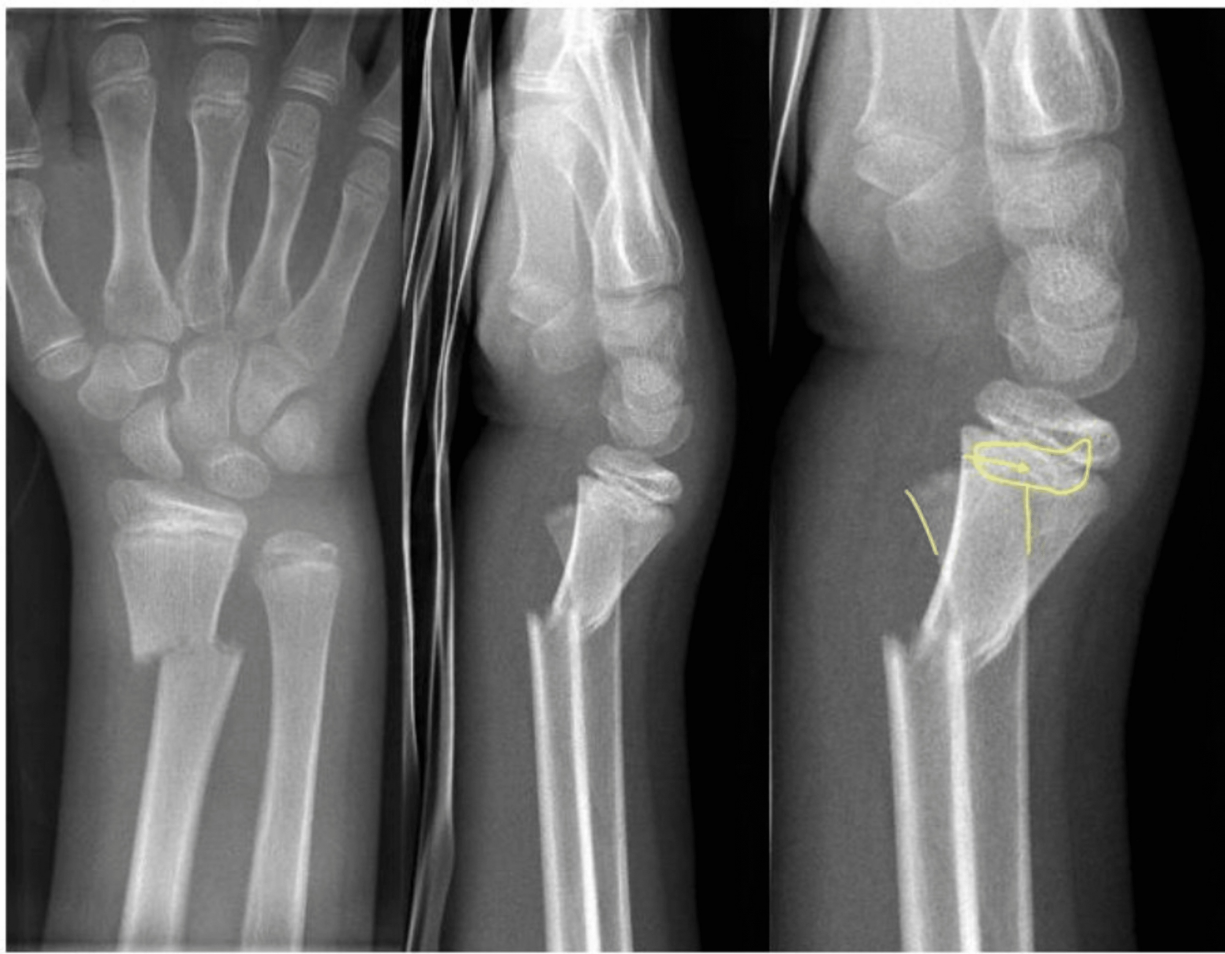
X-rays before surgery X-rays (anteroposterior and lateral view) with distal radial fracture and disruption of the distal ulnar physis. Yellow: epi-metaphyseal relation.

An 11-year-old boy fell onto his extended right wrist while playing soccer. Following the fall, he visited our emergency department, complaining of severe pain and tenderness in his right, dominant wrist. No immobilization had been applied to the wrist prior to his arrival. X-ray images (anteroposterior and lateral views) revealed a dislocated metaphyseal fracture of the distal radius with a volar apex, along with an additional disruption of the distal ulnar physis, consistent with a Salter-Harris Type I fracture (Figure 1).

**Figure 2:**
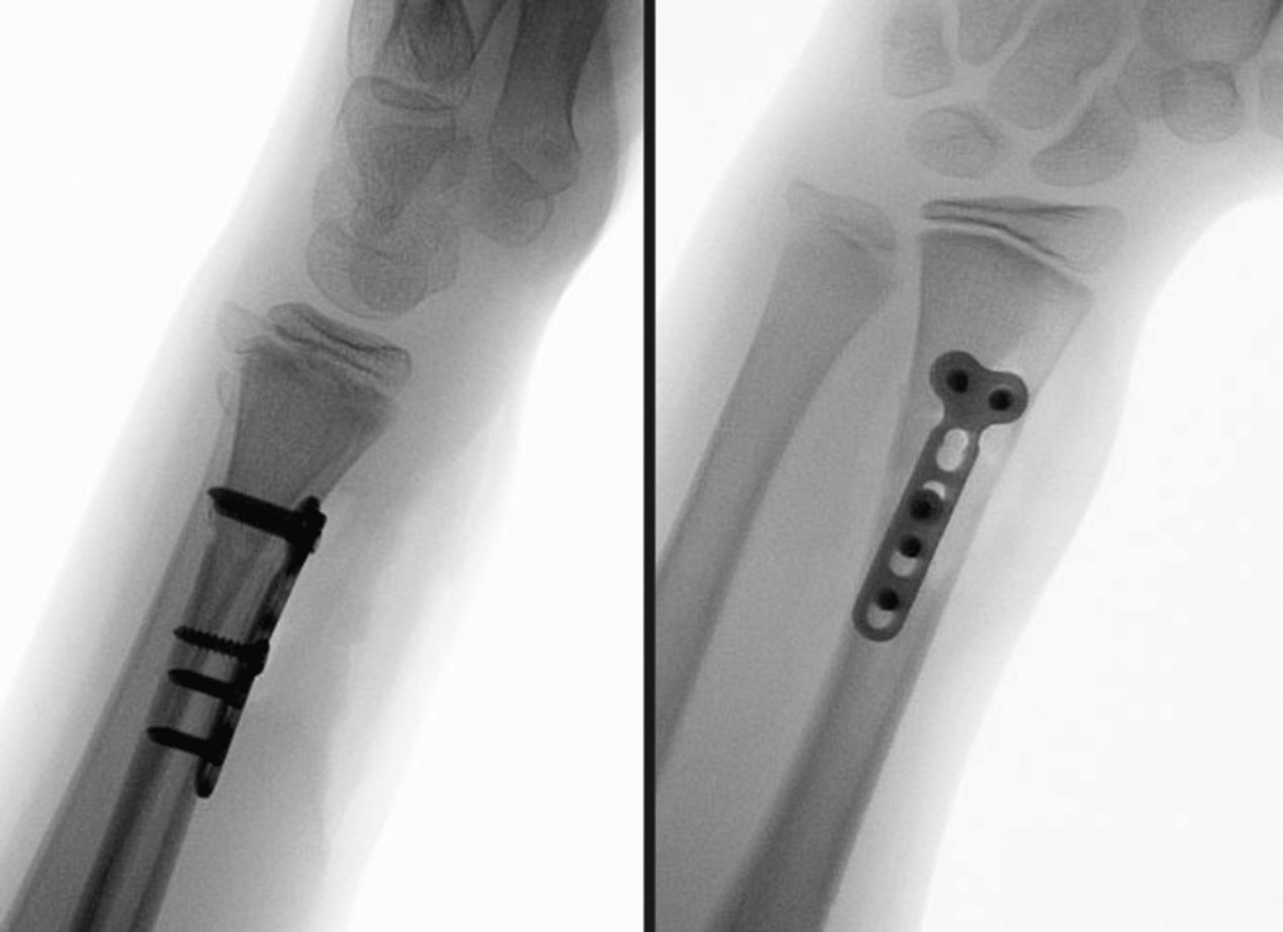
Intraoperative fluoroscopy X-rays of the right forearm (lateral and anteroposterior views) with volar locking plate

After the clinical examination, no neurovascular injury was reported. Primarily the closed reduction under the fluoroscopy was performed; however, the anatomical reposition of the radius could not be achieved. Therefore, the surgery was indicated. The patient and his parents were fully informed about the details of the surgical procedure, its risks, and rehabilitation after the surgery. The open reduction of the radius via modify Henry approach was performed under general anesthesia on the day of injury. Intraoperatively was revealed, that the pronator quadratus muscle was interposed between the fracture fragments, prohibiting reduction. The distal ulnar epiphysis reduced itself once the radius was reduced. Fixation of the radial fracture was achieved by a volar locking plate (Synthes LCP 2.7 mm (Figure 2)).

**Figure 3:**
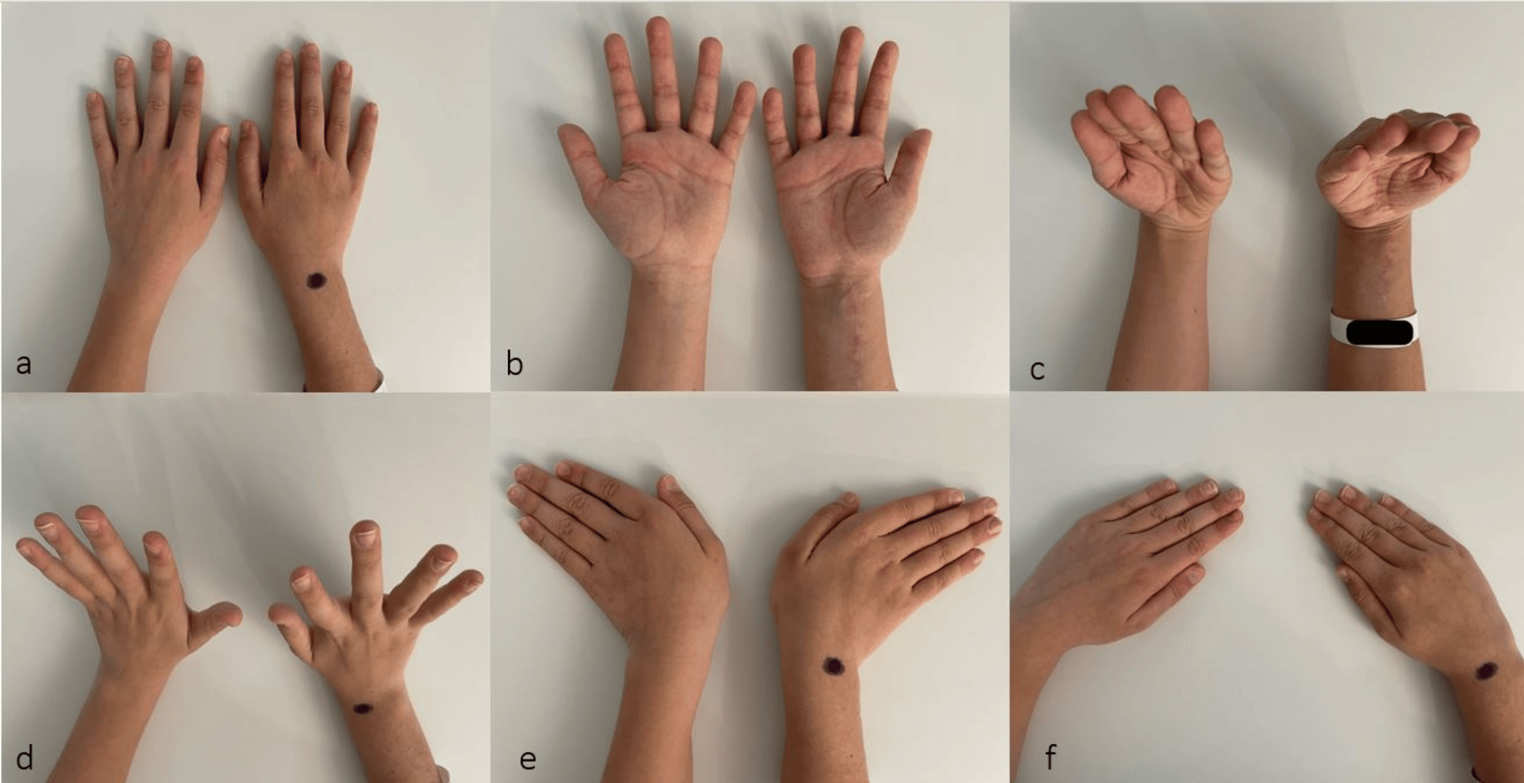
ROM at a six-month follow-up ROM of both hands. The right wrist (operated side) is marked with a black dot. A) Forearm pronation, B) forearm supination, C) wrist flexion, D) wrist extension, E) wrist ulnar abduction, and F) wrist radial abduction ROM, range of motion

To protect the distal ulnar epiphysis from secondary dislocation, a long-arm cast was applied for six weeks. The patient was followed regularly in our outpatient clinic, and no complications were reported by either the patient or his family after surgery. At the six-month follow-up, the patient was pain-free and the wrist's range of motion (ROM) was identical to that of the uninjured left side (Figure 3a-3f).

**Figure 4:**
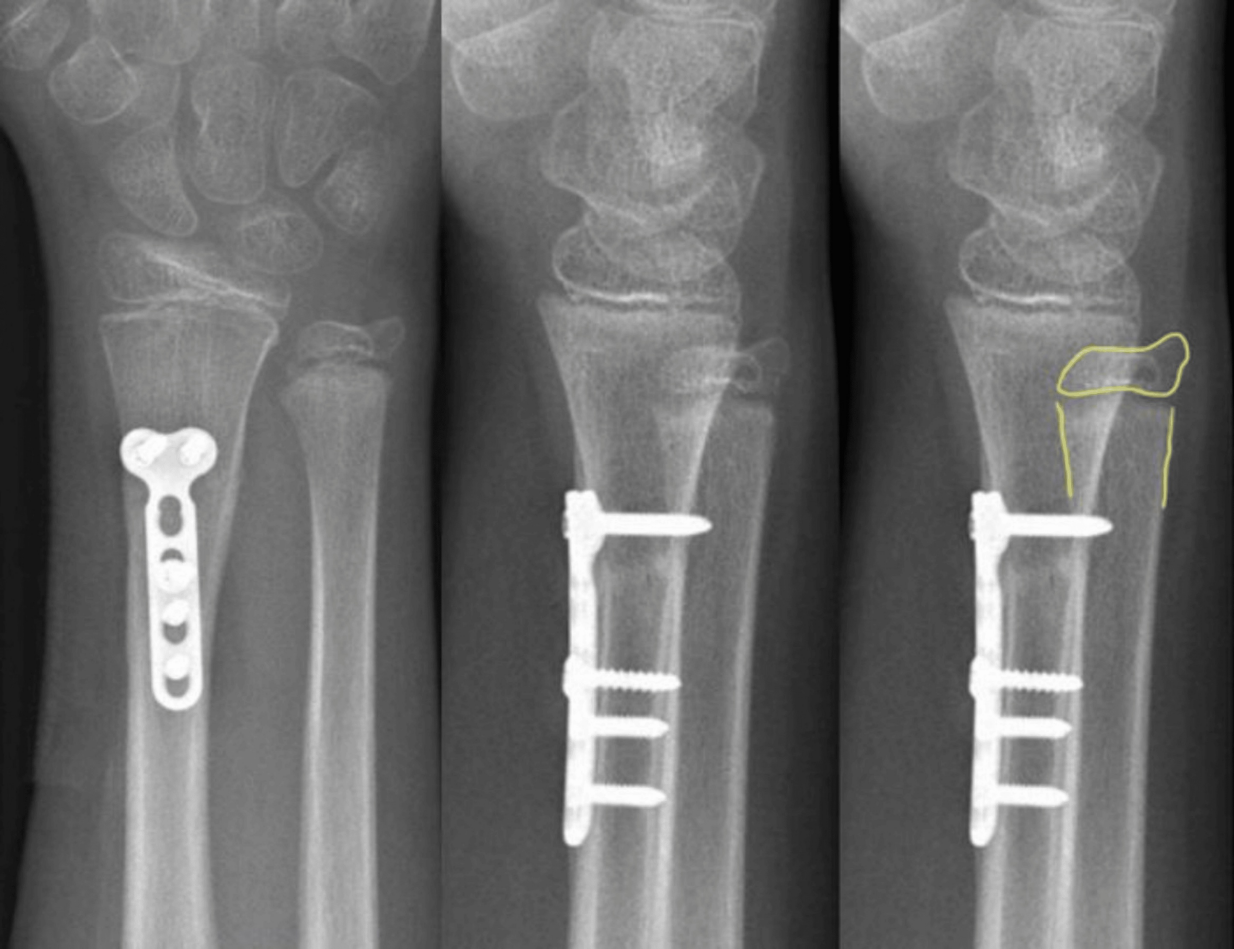
X-rays at six-month follow-up X-rays (anteroposterior and lateral view) with centered distal ulnar epiphysis. Yellow: epi-metaphyseal relation

No instability of the DRUJ was noted during the clinical examination. The distal ulnar epiphysis was well-aligned and the fracture had completely healed (Figure 4).

Patient-reported outcomes were assessed using the PROMIS pediatric upper extremity score, which was 30/30 points at the six-month follow-up (Table 1). 

**Table 1:** PROMIS Pediatric Item Bank v.1.0 - Physical Function - Upper Extremity - Short Form 8a

Pediatric Physical Function - Upper Extremity - Short Form					
In the past 7 days					
	With no trouble	With a little trouble	With some trouble	With a lot of trouble	Not able to do
I could button my shirt or pants	X	-	-	-	-
I could open a jar by myself	X	-	-	-	-
I could open the rings in school binders	X	-	-	-	-
I could pour a drink from a full pitcher	X	-	-	-	-
I could pull a shirt on over my head by myself	X	-	-	-	-
I could pull open heavy doors	X	-	-	-	-
I could put on my shoes by myself	X	-	-	-	-
I could use a key to unlock a door	X	-	-	-	-

Grip strength measured 22 kg, which was comparable to the non-dominant left side (22 kg). Hardware removal (same-day surgery) was performed without any significant complications seven months after the initial surgery. 

## Discussion

Galeazzi-equivalent fractures are very uncommon injuries and only a few cases have been published. The incidence of Galeazzi-equivalent fractures varies considerably from 0.3% to 2.8% [3]. The specific anatomy of the distal radioulnar joint may explain the low incidence of this type of ulnar physeal fracture. The typical mechanism of this type of injury is described as a fall on an outstretched hand in hyperpronation. The peak incidence in the pediatric population occurs between the ages of nine and 13 years [4]. Ogden, et al. [5] offer a possible explanation. TFCC along the meniscus between carpal bones and ulna could absorb some fraction of traumatic force and, thereby, provide extended protection for the ulnar physis. On the other side, the epiphysial plates of children are biomechanically weaker than the ligaments of DRUJ. If an external force is applied to the distal ulna, even without the rupture of the TFCC, isolated avulsion fractures of the epiphyseal plates and Galeazzi-equivalent fractures might occur [6]. In general, closed reduction and above-elbow casting with the hand in maximal supination are sufficient for the treatment of Galeazzi-equivalent fractures with minimal displacement [7,8]. However, failure of closed reduction due to soft tissue interposition requires open reduction.

Several authors [9-11] have documented cases of Galeazzi-equivalent fractures with displacement of various anatomical structures that prevented successful closed reduction, making open reduction necessary. In the case presented here, the closed reduction was unsuccessful due to the interposition of the pronator quadratus muscle between the fracture fragments. To the best of our knowledge, no similar case has been reported in the literature to date.

Even with successful outcomes following either closed or open reduction, complications similar to those seen in other forearm fractures can still arise. These complications may include nonunion, malunion, tendon entrapment, or an infection. A rare but possible complication is median neuropathy, which can result from either direct injury or traction ischemia. Premature physeal closure of the distal ulna is the most feared complication [12,13]. 

Cannata et al. [13] described long-term complications related to radioulnar length discrepancy or styloid nonunion after Salter-Harris Type I-IV injuries. A radioulnar length discrepancy of more than 1 cm occurred in only seven out of 157 patients with distal radial physeal injury (4.4%) but in three out of six patients with distal ulnar physeal injury (50%). All the patients involved in the study follow-up with a radioulnar length discrepancy of less than 1 cm have not complained about pain or worse ROM, even if they were involved in the manual work. 

One of the most concerning complications is premature physeal closure of the distal ulna. This can lead to ulnar minus variance, which results in an imbalance in the length of the radius and ulna. Such imbalanced growth of the distal radius and ulna can disrupt the congruence of the DRUJ and may cause ulnar translation of the carpus. These structural issues can significantly impair wrist function and lead to long-term problems such as pain, reduced ROM, decreased strength, and, in the worst case, dislocation of the DRUJ. The primary limitation in this case is the lack of follow-up after the hardware removal. The patient did not return for subsequent follow-up visits, which hindered a comprehensive assessment of his recovery. As a result, key aspects of rehabilitation and potential issues may have been overlooked or not addressed in a timely manner.

## Conclusions

Galeazzi-equivalent fractures with dislocation of the ulnar epiphysis are exceptionally rare injuries, but they present significant challenges when they do occur. In some cases, these injuries can be treated non-operatively with closed reduction. However, a number of factors, including soft tissue interposition such as periosteal flaps, the tendon of the extensor carpi ulnaris, or, as seen in our case, the pronator quadratus muscle, can make achieving a successful closed reduction very difficult or even impossible. In these instances, the majority of cases will require surgical intervention, typically involving open reduction and internal fixation to restore anatomical alignment and stabilize the fracture. The outcomes following these surgeries are generally satisfactory, with most patients experiencing very good recovery and functional improvement. However, it is critical to inform both the patient and their parents beforehand about the potential risks and complications that can arise postoperatively.

Close monitoring is, therefore, essential. Routine follow-up visits should be scheduled until the patient reaches skeletal maturity to ensure that any problems with bone growth or joint function are detected early. If there is any suspicion of growth abnormalities or disproportionate development of the distal ulna, it is highly recommended that radiographs of the uninjured side are obtained for comparison. This can help identify any subtle asymmetries in bone growth and allow for timely intervention to prevent long-term functional impairment. In conclusion, while surgical intervention for Galeazzi-equivalent fractures with ulnar epiphyseal dislocation is often necessary and can yield positive outcomes, it is essential to maintain vigilant, long-term follow-up care to monitor for complications such as premature physeal closure. Early detection of growth disturbances through regular imaging and clinical evaluation is key to ensuring the best possible functional recovery and preventing significant long-term disability.
